# Lipid Supplement in the Cultural Condition Facilitates the Porcine iPSC Derivation through cAMP/PKA/CREB Signal Pathway

**DOI:** 10.3390/ijms19020509

**Published:** 2018-02-08

**Authors:** Wei Zhang, Hanning Wang, Shaopeng Zhang, Liang Zhong, Yanliang Wang, Yangli Pei, Jianyong Han, Suying Cao

**Affiliations:** 1 The Animal Science and Technology College, Beijing University of Agriculture, Beijing 102206, China; weizhanghwz@gmail.com; 2 State Key Laboratories for Agrobiotechnology, College of Biological Sciences, China Agricultural University, Beijing 100193, China; hanningwang1022@gmail.com (H.W.); sz13060051@cau.edu.cn (S.Z.); i19870503@cau.edu.cn (L.Z.); b1206436@cau.edu.cn (Y.W.); peiyangli@cau.edu.cn (Y.P.); 3 Beijing Advanced Innovation Center for Food Nutrition and Human Health, China Agricultural University, Beijing 100193, China

**Keywords:** fatty acid, porcine iPSCs, reprogramming, mesenchymal–epithelial transition

## Abstract

Large numbers of lipids exist in the porcine oocytes and early embryos and have the positive effects on their development, suggesting that the lipids may play an important role in pluripotency establishment and maintenance in pigs. However, the effects of lipids and their metabolites, such as fatty acids on reprogramming and the pluripotency gene expression of porcine-induced pluripotent stem cells (iPSCs), are unclear. Here, we generated the porcine iPSCs that resemble the mouse embryonic stem cells (ESCs) under lipid and fatty-acid-enriched cultural conditions (supplement of AlbuMAX). These porcine iPSCs show positive for the ESCs pluripotency markers and have the differentiation abilities to all three germ layers, and importantly, have the capability of aggregation into the inner cell mass (ICM) of porcine blastocysts. We further confirmed that lipid and fatty acid enriched condition can promote the cell proliferation and improve reprogramming efficiency by elevating cAMP levels. Interestingly, this lipids supplement promotes mesenchymal–epithelial transition (MET) through the cAMP/PKA/CREB signal pathway and upregulates the *E-cadherin* expression during porcine somatic cell reprogramming. The lipids supplement also makes a contribution to lipid droplets accumulation in the porcine iPSCs that resemble porcine preimplantation embryos. These findings may facilitate understanding of the lipid metabolism in porcine iPSCs and lay the foundation of bona fide porcine embryonic stem cell derivation.

## 1. Introduction

Induced pluripotent stem cells (iPSCs) could be derived from somatic cell by the expression of transcription factors including OCT4, SOX2, KLF4 and c-MYC [1,2]. The iPSCs has become an alternative resource of stem cells for biomedical research and stem cell therapy, without using embryos. Compared with mouse and other model animals, pigs share similarities with human physiology, anatomy and metabolism [3]. Generation of porcine iPSCs may make up for the disadvantages of human iPSCs, such as the limits of in vivo experiment due to ethical issues [4,5]. Thus, the pig could be a promising animal model for studying human disease by providing safety evaluation systems for medicine and performing xenotransplantation.

Currently, porcine iPSCs have been derived from several groups [6,7,8]. However, exogenous genes were hardly silenced in these cell lines and few cell lines contributed to chimeras with germline transmission, demonstrating that the cell culture systems may be not very suitable for the authentic porcine pluripotent stem cells. Large amounts of lipids exist in porcine oocytes and early embryos, which play important roles in oocyte maturation, embryo development and stem cell proliferation [9]. Our results of analysis of the transcriptome of the porcine early embryos at different development stages also suggest that lipid and fatty acid metabolism may be a unique characteristic of porcine pre-implantation embryo development, which may play important roles in the cell fate regulation [4]. Previous reports show that linoleic-acid-induced mouse embryonic stem cells (ESCs) proliferation through Ca^2+^/PKC, PI3K/Akt, and MAPK signaling pathways [10]. Lysophosphatidic acid activated YAP and promotes transgene-free human naive PSCs generation [11]. Sphingosine-1-phosphate and lysophosphatidic acid could promote ESCs proliferation and self-renewal [12,13]. Therefore, we sought to test whether lipid and fatty acids supplement could facilitate porcine iPSCs generation. AlbuMAX is a kind of bovine serum albumin consisting of lipids and fatty acids, which are similar to lipid and fatty acid ingredients in porcine oocytes and early embryos. AlbuMAX not only improved the viability and hatching ability of porcine blastocysts produced in vitro [14], but also regulated human embryonic stem cells (ESCs) self-renewal [15]. Sphere cells that have pluripotency could be induced from mouse fibroblasts in the culture conditions including AlbuMAX [16]. 

In this study, porcine iPSCs were generated and cultured in the conditions containing AlbuMAX. The iPSCs show pluripotency and differentiation potential, and abilities of aggregation into both porcine parthenogenetic blastocysts and mouse early embryos. Genes associated with fatty acid metabolism were upregulated in the iPSCs. In addition, AlbuMAX upregulated cAMP levels and promoted mesenchymal–epithelial transition (MET) through cAMP/PKA/CREB signal pathway, leading to enhance the reprogramming efficiency.

## 2. Results

### 2.1. Fatty Acid Enriched Culture Condition Improves Reprogramming Efficiency

**Figure 1:**
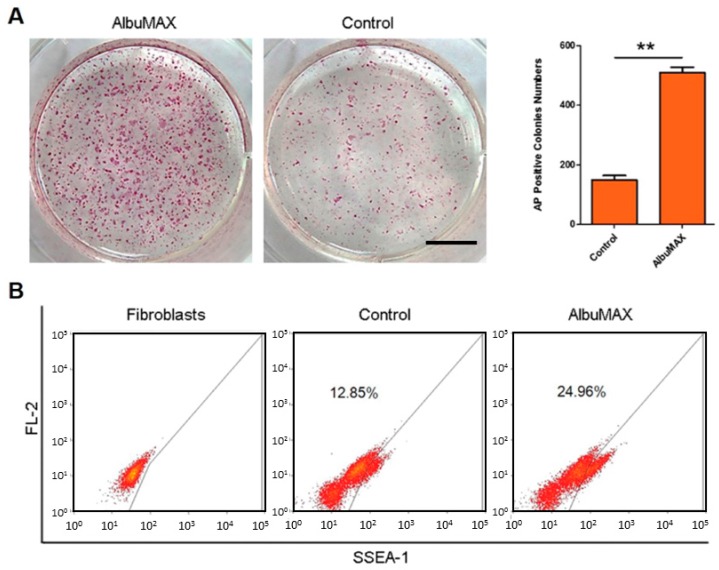
AlbuMAX improves porcine embryonic fibroblasts (PEF) reprogramming efficiency. (**A**) alkaline phosphatase (AP) staining and the numbers of AP-positive colonies. Scale bar, 8 mm. ** *p* < 0.01. (**B**) Analysis of SSEA-1 positive cells by flow cytometry. Fibroblasts were as negative control.

During porcine embryonic fibroblasts (PEF) reprogramming, we counted the numbers of alkaline phosphatase-(AP) positive cells and analyzed the positive cells of pluripotency surface marker SSEA-1. AP-positive colonies in the medium including AlubMAX (called LpiPSCs) were more than that in control group (Figure 1A). Besides, flow cytometry analysis showed that SSEA-1 positive cells proportions were 24.96% which was higher than the control group 12.85% (Figure 1B). These results demonstrate that AlbuMAX could enhance the PEF reprogramming.

### 2.2. The iPSCs Have Pluripotency, Differentiation Potential and Embryo Aggregation Abilities

**Figure 2:**
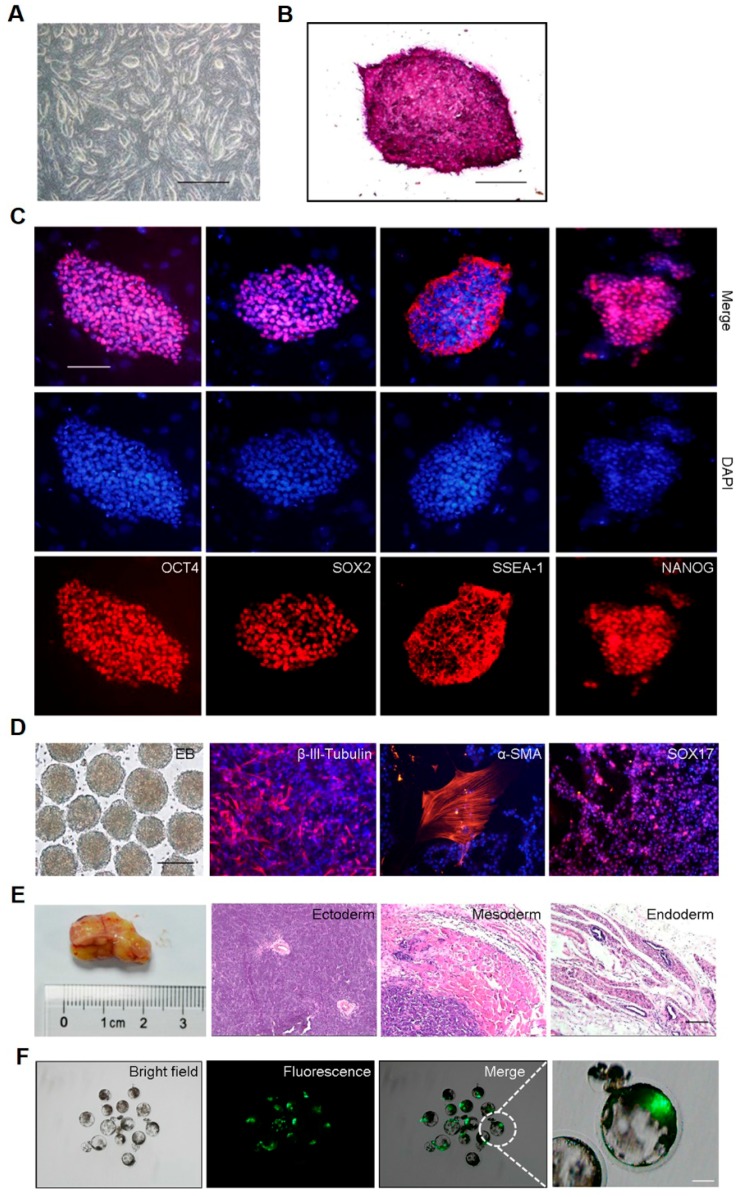
Pluripotency and differentiation potential of LpiPSCs. (**A**) Colonies of LpiPSCs. Scale bar, 500 μm. (**B**) AP staining of LpiPSCs. Scale bar, 100 μm. (**C**) Immunofluorescence staining of pluripotency markers (Red) including OCT4, SOX2, SSEA-1 and NANOG. Nuclei were stained with Hoechst 333342 (Blue). Scale bar, 100 μm; (**D**) Embryoid bodies and immunofluorescence staining of three germ layers. Nuclei were stained with Hoechst 333342 (Blue). Scale bar, 200 μm. (**E**) Teratoma and H&E staining. Scale bar, 200 μm; (**F**) Aggregation of LpiPSCs into inner cell mass (ICM) of porcine parthenogenetic embryos. Scale bar, 100 μm.

The LpiPSCs that were morphologically similar to mouse ESCs were domed and three-dimensional (Figure 2A). In order to examine the pluripotency of LpiPSCs, we performed the AP staining and immunofluorescent staining. These piPSC lines were cultured for more than 10 passages before characterization. The results showed that LpiPSCs were AP-positive (Figure 2B) and expressed pluripotency markers, such as OCT4, SOX2, SSEA-1 and NANOG (Figure 2C). Additionally, in vitro differentiation experiment indicated that LpiPSCs were able to form embryoid bodies (EBs) and differentiate into three germ-layers including endoderm, mesoderm and ectoderm (Figure 2D). In vivo experiment showed that teratoma, which contained three germ-layer cells, could be formed after injection of LpiPSCs in immunodeficient mice (Figure 2E). These results indicate that LpiPSCs have differentiation abilities in vitro and in vivo.

To test the capability of aggregation of LpiPSCs into porcine embryos, we injected LpiPSCs into porcine parthenogenetic embryos at the eight-cell stage and examined when embryos developed to the blastocyst stage. The results indicated that LpiPSCs with green fluorescence incorporated into inner cell masses (ICM) of porcine embryos (Figure 2F). Cross-species chimeric mouse embryos at E8.5–E10.5 developmental stages were generated following injection of naïve human ESCs into mouse morulas [17]. In our work, LpiPSCs were aggregated with two mouse embryos without zona pellucida at the eight-cell stage in microwells (Figure S1A). The reconstructed blastocysts contained LpiPSCs with green fluorescence, indicating that iPSCs were incorporated into mouse blastocysts (Figure S1B). After transplantation of reconstructed embryos into the pseudopregnant mouse, no green fluorescence was detected at E13.5 mouse embryos (Figure S1C). These results suggest that the LpiPSCs have embryonic aggregation ability but it is limited for further in vivo development to full term.

### 2.3. AlbuMAX Promotes iPSCs Proliferation

**Figure 3:**
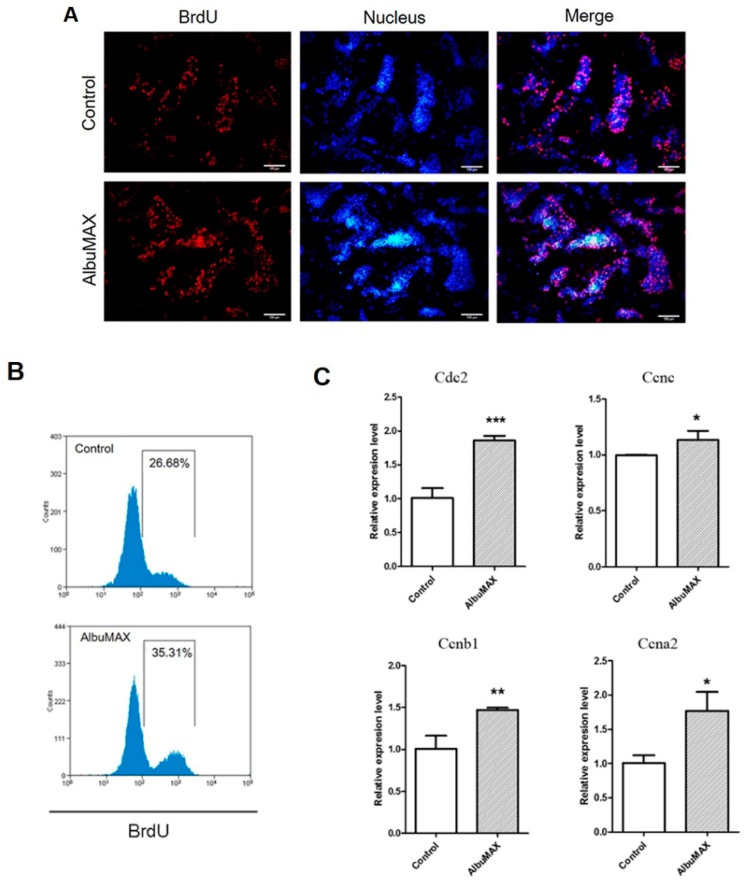
AlbuMAX promotes induced pluripotent stem cells (iPSCs) proliferation. (**A**) Immunofluorescence staining of BrdU (Red) in porcine iPSCs. Nuclei were stained with Hoechst 333342 (Blue). Scale bar, 100 μm; (**B**) Analysis of BrdU positive cells by flow cytometry. * *p* < 0.05, ** *p* < 0.01, *** *p* < 0.001. (**C**) Quantitative RT-PCR analyzed the expression of cell cycle related genes.

As shown in AP staining during reprogramming, more AP-positive cells were in LpiPSCs than the control. Therefore, AlbuMAX may promote cell proliferation. In order to confirm it, we performed the Bromodeoxyuridine (5-bromo-2'-deoxyuridine, BrdU) immunofluorescent staining and flow cytometry analysis of BrdU positive cells in iPSCs. The results revealed that a lot of BrdU positive cells existed in LpiPSCs. The proportions were 35.31% (Figure 3A,B). As a control, 26.28% of BrdU positive cells were detectable (Figure 3A,B). Quantitative RT-PCR showed that genes related with cell cycle including Cdc2, Ccnc, Cdc20 and Ccna2 were highly expressed in LpiPSCs (Figure 3C). These findings suggest that AlbuMAX improved the PEF reprogramming efficiency through promoting cell proliferation. A high proliferation rate was a key factor for mouse and human iPSCs generation [18,19].

During reprogramming, *Nanog* and *EpCAM* expression levels increased as compared to the control group (Figure S2B) while expression levels of pluripotency genes, such as Oct4, Sox2, Sall4, Cdh1 and Esrrb, were not obviously elevated (Figure S2B). As per previous reports, the exogenous pluripotency genes were not silenced and not reduced completely (Figure S2A), suggesting more experimentation can be done to explore the detail mechanisms. By contrast, when porcine iPSCs derived in medium without AlbuMAX were cultured in the AlbuMAX medium for three and six passages respectively, the endogenous genes were not changed significantly (Figure S2C). These results demonstrate that lipids supplement mainly promotes cell proliferation to improve the efficiency of the porcine iPSCs reprogramming.

### 2.4. AlbuMAX Contributes to Lipid Droplets Accumulation

**Figure 4:**
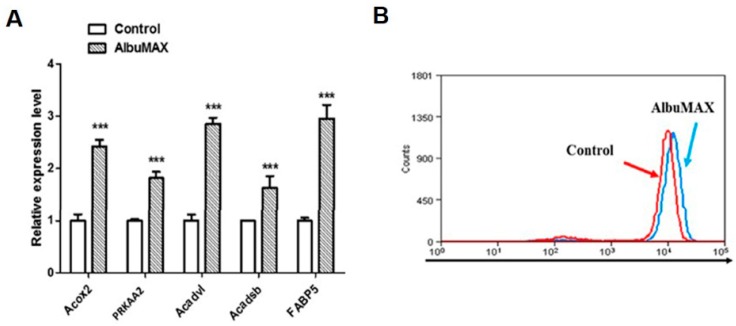
AlbuMAX facilitates lipid droplets accumulation. (**A**) Quantitative RT-PCR analyzed the expression of fatty acid metabolism related genes. *** *p* < 0.001; (**B**) Analysis of lipid droplets content in iPSCs by flow cytometry.

Quantitative RT-PCR showed that genes associated with signaling pathways including fatty acid biosynthesis (i.e., Prkaa2), transport (i.e., Fabp5) and metabolism (i.e., Acadcl, Acadsb) were up-regulated significantly in LpiPSCs (Figure 4A), which were similar to that in porcine pre-implantation embryos [4]. These results suggest that the culture system containing AlbuMAX could increase fatty acid metabolism of porcine iPSCs, as well as, the patterns of fatty acid metabolism-related gene expression and metabolic characteristics in LpiPSCs are similar to that in the porcine early embryos. In order to test lipid droplets content, iPSCs were stained with Nile Red that could specifically stain lipid droplets in cells and then analyzed by flow cytometry. Flow cytometry analysis of lipid droplets content showed that the fluorescent signal of Nile Red in LpiPSCs was higher than the control (Figure 4B), indicating that AlbuMAX facilitated lipid droplets accumulation in the porcine iPSCs. 

### 2.5. AlbuMAX Promotes MET During the Reprogramming of Fibroblasts

**Figure 5:**
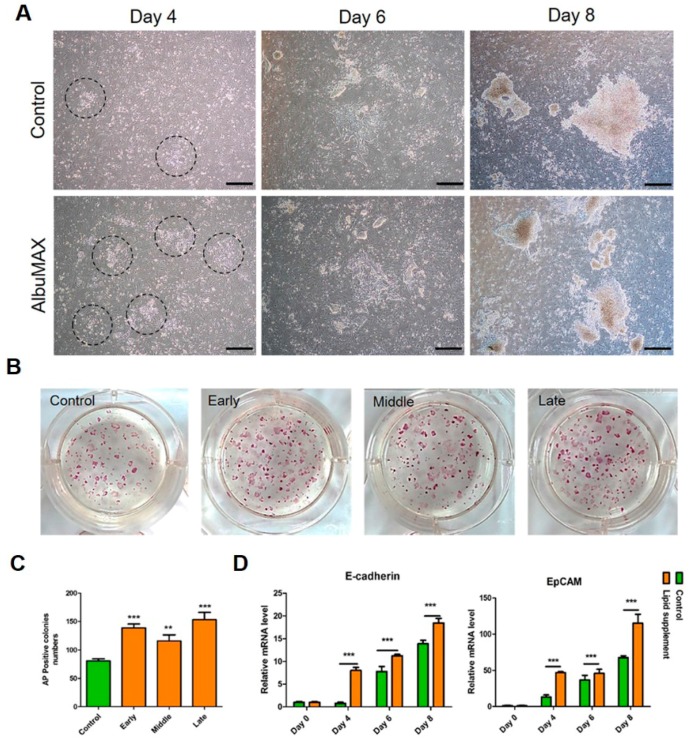
AlbuMAX promotes mesenchymal–epithelial transition (MET) during reprogramming. (**A**) MET process in iPSCs. Black circles indicated cells in the MET process. Scale bar, 500 μm; (**B**) AP staining of iPSCs following treatment of AlbuMAX at different stages; (**C**) Analysis of AP-positive colonies. ** *p* < 0.01, *** *p* < 0.001; (**D**) Quantitative RT-PCR analyzed the expression of *E-cadherin* and *EpCAM* during reprogramming. Day 0, Day 4, Day 6 and Day 8 indicated the time that AlbuMAX was added. *** *p* < 0.001.

MET was known as the first barrier in the reprogramming process [20] and occurred after PEF infected with pluripotency factors [21]. To test whether AlbuMAX participate in the MET, we added AlbuMAX at different stages of reprogramming, such as at the early stage (Day 3–4), at the middle stage (Day 5–6) and at the late stage (Day 7–8). More cells with morphology change were in the AlbuMAX group than the control, as black circles indicated (Figure 5A). AP-positive colonies in the AlbuMAX medium were more than in the control at the early, middle and late stages (Figure 5B,C). In addition, quantitative RT-PCR revealed that MET markers including *E-cadherin* and *EpCAM* were upregulated significantly at different stages (Figure 5D), demonstrating that AlbuMAX promoted expression of genes associated with MET. The data indicated that fatty acid metabolism play an important role in the whole process of reprogramming.

### 2.6. AlbuMAX Enhances Reprogramming via Activating cAMP/PKA/CREB Signaling Pathway

**Figure 6:**
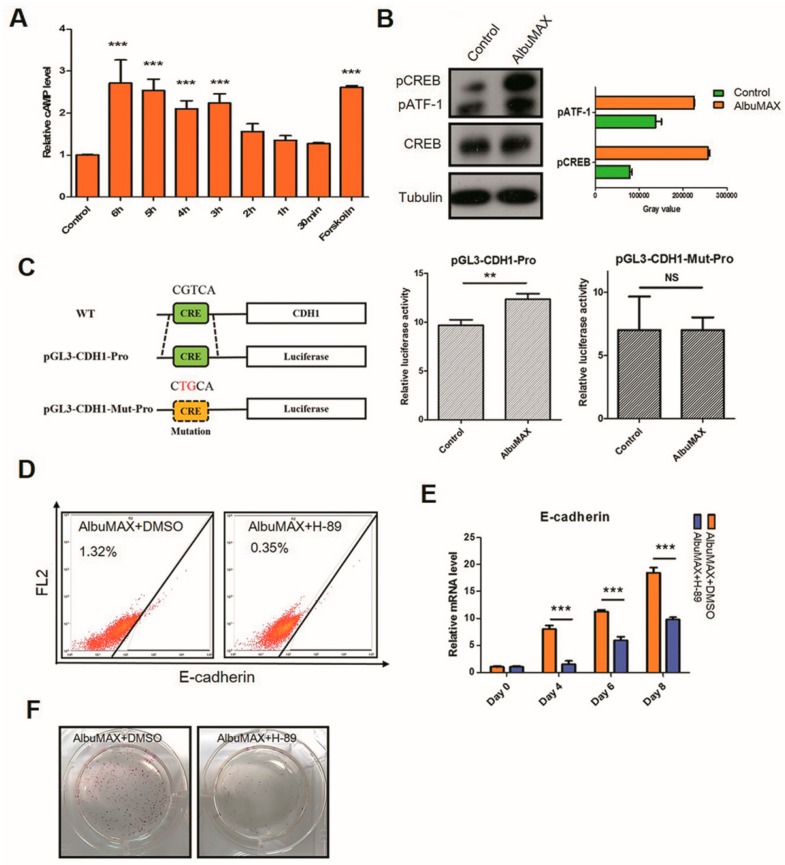
AlbuMAX enhances reprograming through cAMP/PKA signaling pathway. (**A**) Analysis of cAMP levels after AlbuMAX treatment at different time. The control was treated with 1% BSA for 30 min. The positive control group was treated with Forskolin (10 μM) for 30 min. *** *p* < 0.001; (**B**) Western blot detected the phosphorylation of CREB and Activating transcription factor-1 (ATF-1); (**C**) Luciferase reporter and mutation reporter construction and luciferase activity assay. ** *p* < 0.01; (**D**) E-cadherin expression level analysis by flow cytometry; (**E**) Quantitative RT-PCR analyzed the expression of *E-cadherin* during reprogramming. Day 0, Day 4, Day 6 and Day 8 indicated the time that H-89 (20 μM) was added. *** *p* < 0.001; (**F**) AP staining during reprogramming.

Multiple fatty acids and phospholipids are the agonists of G-protein-coupled receptors (GPCRs), such as both sphingosine-1-phosphate and lysophosphatidic acid, which may activate the downstream cAMP/PKA signaling pathway [22]. We hypothesize that the cAMP/PKA pathway is active in LpiPSCs and AlbuMAX may activate the signaling pathway. To confirm the hypothesis, we examined cAMP levels after treatment of PEF in the AlbuMAX medium. The results showed that cAMP levels in PEF after treatment for 4–6 h were more than twice in the control group, which were similar to that after treatment with cAMP activator Forskolin (Figure 6A). Western blot assay also indicated that the phosphorylation levels of CREB and activating transcription factor (ATF) that are transcription factors downstream of cAMP/PKA pathway elevated after AlbuMAX incubation (Figure 6B). These data demonstrate AlubMAX could activate the cAMP/PKA signaling pathway during porcine PEF reprogramming into iPSCs.

Transcription factor CREB contains conserved binding sites in the regulatory regions of *E-cadherin* promoter. Therefore, we constructed a luciferase reporter and the mutation vector of *E-cadherin* promoter containing CREB binding sites (Figure 6C). The luciferase activity assay showed that luciferase expression levels following supplementation with AlbuMAX in the medium were significantly more elevated than the control, while the luciferase levels of mutation treated with AlbuMAX did not increase much more than the control group (Figure 6C). These data demonstrate that AlbuMAX could activate CREB which binds to *E-cadherin* promoter regulatory regions and then regulate *E-cadherin* expression.

To further confirm the cAMP/PKA signaling pathway play a role, the PKA inhibitor H-89 was added in the cell culture systems After which *E-cadherin* positive cells decreased from 1.32% to 0.35% (Figure 6D). *E-cadherin* expression also downregulated significantly via quantitative RT-PCR detection (Figure 6E). To test the PEF reprogramming efficiency in the culture conditions including PKA inhibitor, AP staining was performed, showing that AP-positive colonies were reduced significantly (Figure 6F). These results indicate that AlbuMAX is impeded by inhibiting cAMP/PKA signaling pathway.

**Figure 7:**
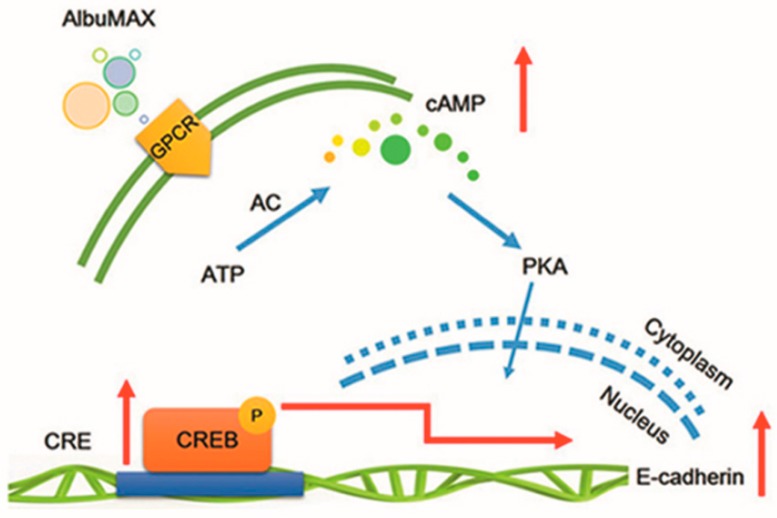
Schematic diagram of AlbuMAX working model in reprogramming.

Taken together, AlbuMAX acts as the GPCRs’ agonist and mainly participates in the reprogramming through cAMP/PKA signaling pathway. AlbuMAX upregulates cAMP levels, as well as, PKA improves CREB phosphorylation levels and then CREB binds to regulatory regions of *E-cadherin* to promote *E-cadherin* expression. MET process is enhanced and reprogramming efficiency is thus improved, as shown in the model (Figure 7).

## 3. Discussion

Porcine oocytes and embryos at early stages contain a lot of lipids including triglyceride, cholesterol, free fatty acids and phospholipids, wherein the content is higher than that of the mouse, bovine and ovine oocytes [9]. Lipids facilitate oocyte maturation, embryo development and cell proliferation [14,23,24]. Lipids may thus contribute to the generation of porcine iPSCs. However, lipids in porcine oocytes are complicated and the metabolites could be transformed. As a result, it is difficult to confirm what kind of lipid has a function. Therefore, AlbuMAX containing triglyceride, cholesterol, free fatty acids and phospholipids could simulate the lipid niche in porcine oocytes and early embryos well. In our study, we identify AlbuMAX that motivates PEF reprogramming. The porcine iPSCs derived from PEF in AlbuMAX medium were pluripotent and had differentiation ability and embryo aggregation ability.

Early studies demonstrated that a high proliferation rate was important for cell reprogramming in both mouse and human [18,19]. Our study suggested that acceleration of cell cycle progression and enhancement of cell proliferation were also required for porcine iPSCs generation.

Promotion of MET process significantly enhances reprogramming [25]. Overexpression of MET marker *E-cadherin* based on the traditional transcription factors promoted MET and as a result improves reprogramming. *E-cadherin* could even replace Oct4 to induce mouse somatic cell reprogramming [26]. *E-cadherin*-mediated cell–cell contact is also critical for induced pluripotent stem cell generation. Knockdown *E-cadherin* reduced reprogramming efficiency [27]. One notable finding in our work is that AlbuMAX works in PEF reprogramming through upregulating *E-cadherin* and promoting MET. High performance liquid chromatography analysis demonstrates that ingredients of AlbuMAX are albumin and lipids including triglyceride, cholesterol, free fatty acids and phospholipids. Lipids but not albumin in AlbuMAX are responsible for stimulating ESCs renewal [15]. Phospholipids that are GPCRs agonists but not free fatty acids promotes ESCs renewal [15,22]. Triglyceride works primarily as an energy substance and cholesterol is the main ingredient of cell membrane. Therefore, phospholipids in AlbuMAX may play leading roles in stimulating the MET process via elevating cAMP levels and then activating downstream transcription factors through PKA pathway.

Taken together, AlbuMAX added in cell medium had positive effects on iPSCs generation. Despite the fact AlbuMAX could facilitate reprogramming in a pig, endogenous pluripotency genes were inadequate to support porcine iPSCs maintenance and exogenous genes were still expressed to maintain iPSCs pluripotency. Key pluripotency regulators in porcine iPSCs may be different from conventional reprogramming factors and culture conditions in mouse and human iPSCs. Optimal induced factors, chemical compounds and cytokines supplemented in culture systems need to be explored so as to find the methods of activating endogenous pluripotency genes and then silencing exogenous genes during porcine somatic reprogramming.

## 4. Materials and Methods

### 4.1. Animal Experiments

Immunodeficient mice were purchased from Vital River Laboratories (Beijing, China) and used for the teratoma assay. The animal study proposal (Approved code: SKLAB-2013-04-01, 1 April 2013) in this study were approved by the Animal Care and Use Committee of China Agricultural University.

### 4.2. Generation of Porcine iPSCs and Cell Culture

Retroviral virus vectors (pMXs vector) separately carrying porcine Oct4, Sox2, Klf4 and c-Myc were used to generate iPSCs. The viral production was performed as the method described [28]. Retroviruses were used to infect PEF containing Actin-ZsGreen for 12 h. Cells were passaged and seeded on the mitomycin-treated mouse embryonic fibroblasts (also called feeder cells) following two rounds of infection. Then the medium was changed to reprogramming medium on the second day. The medium was changed every two days. The efficiency of PEF reprogramming was evaluated by AP staining. Colonies were picked up at about Day 30. 

The reprogramming medium and the porcine iPSCs medium contained Dulbecco's Modified Eagle Medium (DMEM), 15% fetal bovine serum (FBS) (Gibco, Waltham, MA, USA), nonessential amino acids, Gluta-MAX, penicillin/streptomycin (all from Gibco), β-mercaptoethanol (Sigma, Lenexa, KS, USA), human LIF, 2i (CHIR99021 and PD0325901) (Selleck, Houston, TX, USA) and 1% AlbuMAX (called AlbuMAX medium). The medium in the control group contained 15% fetal bovine serum (FBS), nonessential amino acids, Gluta-MAX, penicillin/streptomycin, β-mercaptoethanol (Sigma), human LIF, 2i (CHIR99021 and PD0325901) (Selleck) and 1% BSA (called control medium). The iPSCs were passaged by TrypLE at a ratio of 1:6 every 2–3 days.

For embryoid body formation, the iPSCs were digested and maintained in the medium mentioned above removal of human LIF, 2i (CHIR99021 and PD0325901) and AlbuMAX on a shaker (40 r/min) in a CO_2_ incubator.

### 4.3. Quantitative RT-PCR

Total RNA was extracted using a Qiagen RNeasy mini RNA kit (Qiagen, San Diego, CA, USA) and then reverse-transcribed by Oligo-dT primer and M-MLV Reverse Transcriptase (Promega, Fitchburg, WI, USA). The cDNAs were used for quantitative RT-PCR analysis with LightCycler 480 SYBR Green I Master Kit (Roche, Basel, Switzerland). The data was analyzed using the comparative CT method. 

The primers were as bellows:

**Forward (5′ to 3′)****Reverse (5′ to 3′)****References**pMXs-Oct4GACGGCATCGCAGCTTGGATACACGAGAAGGCGAAGTCGGAAG[29]pMXs-Sox2GACGGCATCGCAGCTTGGATACACGGCTGTTCTTCTGGTTGC[29]pMXs-Klf4GACGGCATCGCAGCTTGGATACACGTCTTTGCTTCATGTGGG[29]pMXs-MycGACGGCATCGCAGCTTGGATACACGAAATAAGGCTGCACCGAGT[29]NanogCATCTGCTGAGACCCTCGACGGGCTTGTGGAAGAATCAGG[29]EF-1αAATGCGGTGGGATCGACAAACACGCTCACGTTCAGCCTTT[29]Endo-Oct4CAAACTGAGGTGCCTGCCCTTCCAAACTGAGGTGCCTGCCCTTC[29]Endo-Sox2CATCAACGGTACACTGCCTCTCACTCTCCTCCCATTTCCCTCTTT[29]Endo-Klf4CATGAGTTGGGGGAGGGAAGACTCACCAAGCACCATCGTT[29]Endo-MycATCCAAGACCACCACCACTGATCCAAGACCACCACCACTG[29]Cdh1TGGGCCGAGTGAGTTTTGAATGACTGTAACCACACCGTCG[29]Sall4ATCGACGTTTATCCGAGCCCATCGACGTTTATCCGAGCCC[29]EpCAMTGCTCTTTGAATGCGCTTGGAGAGCCCATCGTTGTTCTGG
EsrrbCCGGACAAACTCTACGCCATGCTTGGCCCAACCAATGATG
Cdc2TTTTCAAAGCTGGCTCTGGGAGGGATGTGGTAGATCCCAGCTTA
CcncAGAAAGATGCCAGACGGTGGAGGAGGTTTTGGTTTCGGCA
Ccnb1AGATCGCAGCAGGAGCTTTTCCTCGATTCACCACGACGAT
Ccna2CTAACATTGCAGCAGACGGCATCCTTAAGAGGCGCAACCC
Acox2AGGACTCAGGACGAGACACATTGAAGGACGGCATGCATCT
Prkaa2ATTCTGTCACTGCGGAGAGCAATCCATGGTGTGACTGCCC
AcadvlGAAGTCAAATGCCTGCCAGCATGTTGGCGCTCACCATGTA
AcadsbACACCAAGTGGCTCATACGGTACCAATCTTCGCGTCTCGG
Fabp5AGGCACCAGTCCGCTTATTCTTTCGTAGGGCCATTCCCAC


### 4.4. Alkaline Phosphatase Staining and Immunofluorescence Staining

The alkaline phosphatase (AP) staining was performed using an Alkaline Phosphatase Detection Kit (Millipore, Temecula, CA, USA) according to the manufacturer’s instructions.

For immunofluorescence staining, cells were fixed in 4% PFA for 20 min, permeabilized with 0.2% Triton X-100 for 15 min, and blocked in the blocking buffer (Beyotime, Shanghai, China) for 1 h. Primary and secondary antibodies were incubated overnight at 4 °C and for 1 h at room temperature, respectively. Membrane proteins were not permeabilized in the 0.2% Triton X-100. Nuclei were stained with Hoechst 333342 for 2 min at room temperature. The primary antibodies were as follows: OCT4 (Santa cruz, sc-5297, Dallas, TX, USA), SOX2 (Abcam, ab97959, San Francisco, CA, USA), SSEA-1 (Cell Signaling Technology, 4744S, Danvers, MA, USA), Nanog (Peprotech, 500-P234, Rocky Hill, CT, USA), SOX17 (Cell Signaling Technology, 81778), α-SMA (Abcam, ab5694), β-III-TUBULIN (Abcam, ab18207) and BrdU (Abcam, ab152095). The secondary antibodies used here were as follows: goat anti-rabbit Alexa Fluor 594 (Invitrogen, A11037, Waltham, MA, USA), goat anti-mouse Alexa Fluor 594 (Invitrogen, A21044), goat anti-mouse Alexa Fluor 594 (Invitrogen, A11032), and goat anti-chicken Alexa Fluor 594 (Invitrogen, A11042).

For lipid droplets staining using Nile Red (Sigma, N-1142). The cells were fixed with 4% PFA for 20 min at room temperature and washed three times with Dulbecco's Phosphate Buffered Saline (DPBS) and then incubated with 5 ng/ml Nile Red for 5 min at room temperature.

### 4.5. Flow Cytometry Analysis

For flow cytometry analysis, single cells after digestion of colonies with Tryple were stained with primary antibody and secondary antibody for 1 h, respectively. These cells were then washed three times with DPBS and filtered through a 40-μm falcon. Fluorescence activated Cell Sorting (FACS) analysis was performed using the Moflo-XDP (Beckman, Indianapolis, IN, USA).

### 4.6. Teratoma Formation Assay

For teratoma formation, the porcine iPSCs (2 × 10^7^) were subcutaneously injected into the BALB/c nude mice. After two months, teratoma were harvested for histological analysis by hematoxylin and eosin (H&E) staining. For H&E staining, nuclei were stained with the alum hematoxylin and eosinophilic structures were stained with eosin.

### 4.7. Parthenogenetic Embryo Injection

The maturation and parthenogenetic activation (PA) of porcine oocytes were performed as described [30]. Embryos at eight-cell stage were selected for the injection assay. About 15–20 porcine iPSCs were injected into every embryo by micromanipulation. The injected embryos were then transferred into the Porcine Zygote Medium-3 (PZM-3) for 3–4 days. The numbers of blastocysts and chimeric blastocysts were then analyzed.

### 4.8. Western Blot

Cells were washed in cold DPBS and then lysed in sodium dodecyl sulfate (SDS) sample buffer on ice for 30 min. After centrifugation at 10,000× *g* for 15 min (4 °C), supernatant was collected. The protein concentration of samples was measured using the BCA Protein Assay Kit (Beyotime). The samples were boiled at 99 °C for 5 min and were transferred on ice. The denatured protein samples were used for Sodium dodecyl sulfate polyacrylamide gel electrophoresis and then transferred to polyvinylidene fluoride (PVDF) membranes. The PVDF membranes were in 5% nonfat milk for blocking and probed overnight at 4 °C with primary antibodies. Secondary antibodies were then probed for 1 h at room temperature. Protein bands were finally detected by SuperSignal West Dura Extended Duration Substrate (Thermo Scientific, Waltham, MA, USA).

### 4.9. Detection of cAMP Levels

The cAMP levels of cells were detected using the cAMP-Glo assay kit according to the protocol (Promega). Cells were treated with an agonist or in the induction buffer for the desired length of time and then lysis buffer was added. The plate was incubated with shaking for 15 min. The cAMP detection solution was prepared and added to all wells, mixed and incubated for 20 min. Kinase-Glo^®^ reagents were supplemented into all reactions and incubated for 10 min. The luminescence measurement values were used to calculate the cAMP concentration according to the kit manual.

### 4.10. The Luciferase Reporter Construction and Luciferase Activity Analysis

CREB binding cites in the *E-cadherin* promoter were synthesized directly as shown below:
Top chaintcgagACTCTGTCTCAGTTATTTTTCCCT**CGTCA**AGAGCCATCTGAAGGAGAGGCaBottom chainagcttGCCTCTCCTTCAGATGGCTCTTGACGAGGGAAAAATAACTGAGACAGAGTc

Bases in lower-case were the cohesive ends. Bases in upper-case were the binding sites. In mutation vector construction, mutation cites were designed in the primers and mutation vectors were amplified by rolling circle amplification. CGTAC were transformed to CTGCA in mutation vectors.

The primers were shown as below:
ForwardAGTTATTTTTCCCT**CTGCA**AGAGCCATCTReverseAGATGGCTCTTGCAGAGGGAAAAATAACT

Luciferase activity analysis was performed following cell transfection 24 h using the Dual-Glo Luciferase Assay System (Promega).

## Supplementary Materials

Supplementary materials can be found at http://www.mdpi.com/1422-0067/19/2/509/s1.
