# Supplementary material for: Lipid Supplement in the Cultural Condition Facilitates the Porcine iPSC Derivation through cAMP/PKA/CREB Signal Pathway
Source: Int J Mol Sci. 2018 Feb 8;19(2):509. doi: 10.3390/ijms19020509 (PMC5855731; doi:10.3390/ijms19020509)
Supplement: Supplementary file 1 [file ijms-19-00509-s001.pdf]

Supplementary Information

A

B

C

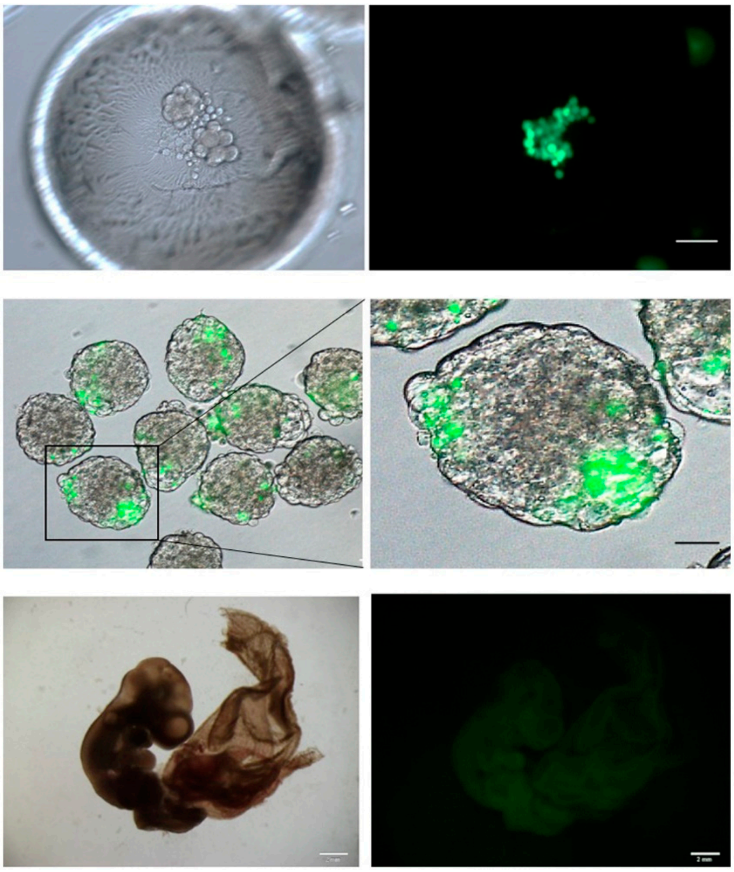

Figure S1. Aggregation of porcine iPSCs with mouse embryos. (**A**) Aggregation of LpiPSCs with two mouse embryos at 8-cell stage in micro wells. Green fluorescence represents LpiPSCs. Scale bar, 200 μm. (**B**) Reconstructed blastocysts. Green fluorescence represents LpiPSCs. Scale bar, 50 μm. (**C**) Reconstructed embryos at E 13.5. No green fluorescence was detected. Scale bar, 2 mm.

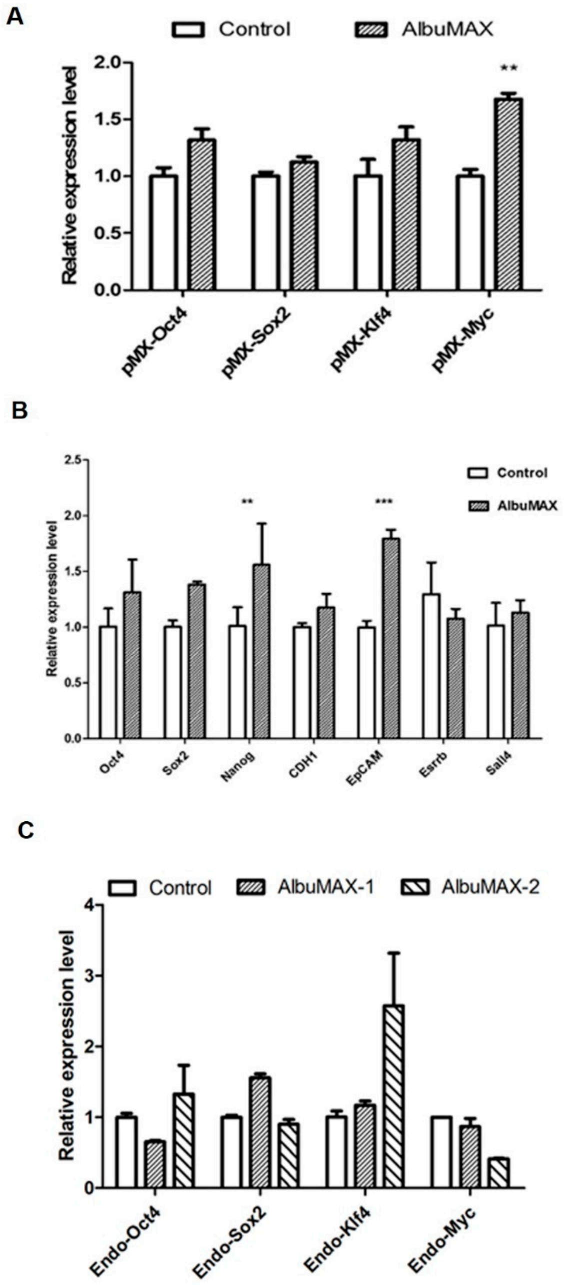

Figure S2. Examination of gene expression by quantitative RT-PCR. (**A**) Quantitative RT-PCR analyzed the expression of exogenous genes in LpiPSCs. ** $p < 0.01$. (**B**) Quantitative RT-PCR analyzed the expression of pluripotency genes in LpiPSCs. ** $p < 0.01$; *** $p < 0.001$. (**C**) Gene expression of endogenous genes in porcine iPSCs. AlbuMAX-1 and AlbuMAX-2 represent porcine iPSCs derived in medium without AlbuMAX were cultured in the AlbuMAX medium for three and six passages respectively.
